# Temporal σ^B^ stress-response profiles impact *Bacillus subtilis* fitness

**DOI:** 10.1128/msphere.00719-23

**Published:** 2024-01-18

**Authors:** Sidney R. Bush, Shelby Sanders, Nicholas Frey, Christopher W. Hamm, Madeline Toews, Sarah Winburn, Emily J. Fayard, AnaLisa Rodriguez, Nicholas S. Boyne, Jacob S. Osborne, Matthew T. Cabeen

**Affiliations:** 1 Department of Microbiology and Molecular Genetics, Oklahoma State University, Stillwater, Oklahoma, USA; The University of Iowa, USA

**Keywords:** *Bacillus subtilis*, sigma factors, stress response, fitness, competition

## Abstract

The Gram-positive model organism *Bacillus subtilis* responds to environmental stressors by activating the alternative sigma factor σ^B^. The sensing apparatus upstream of σ^B^ activation is thought to consist of cytoplasmic stressosomes—megadalton-sized protein complexes that include five paralogous proteins known as RsbRs. The RsbRs are presumed to be involved in stress sensing and the subsequent response. Perturbations to the RsbR complement in stressosomes by engineering cells that produce only one of the RsbR paralogs (“single-RsbR strains”) lead to altered σ^B^ response dynamics with respect to timing and magnitude. Here, we asked whether such changes to σ^B^ response dynamics impact the relative fitness of a strain. We competed strain pairs with different RsbR complements under ethanol and sodium chloride stress and found not only differences in relative fitness among wild-type and single-RsbR strains but also different relative fitness values in the two different stressors. We found that the presence of RsbRA, which dominates the wild-type σ^B^ response, enhances fitness in ethanol but is detrimental to fitness in NaCl. Meanwhile, RsbRD-only cells were among the most fit in NaCl. Strains producing hybrid RsbR fusion proteins displayed different fitness values that depended on the RsbR proteins from which they were derived. Our results here suggest that σ^B^ response dynamics can impact fitness, highlighting the physiological importance of the unusual stressosome-based general stress response system of *B. subtilis*.

**IMPORTANCE:**

The model bacterium *Bacillus subtilis* uses cytoplasmic multiprotein complexes, termed stressosomes, to activate the alternative sigma factor σ^B^ when facing environmental stresses. We have previously shown that genetically manipulating the complement of putative sensor proteins in stressosomes can alter the dynamics of the σ^B^ response in terms of its magnitude and timing. However, it is unknown whether these response dynamics impact the fitness of cells challenged by environmental stressors. Here, we examine the fitness of strains with different σ^B^ responses by competing strain pairs in exponential-phase co-cultures under environmental stress. We find that strains with different response dynamics show different competitive indices that differ by stressor. These results suggest that the dynamics of the σ^B^ response can affect the fitness of cells facing environmental stress, highlighting the relevance of different σ^B^ dynamics.

## INTRODUCTION

The Gram-positive model bacterium *Bacillus subtilis* and a few closely related species, such as *Listeria monocytogenes*, respond to environmental stressors by activating the alternative sigma factor σ^B^ in a process known as the general stress response (GSR) (1–3). The GSR in *Bacillus* and *Listeria* is distinguished from environmental stress-response pathways in other bacteria by the presence of cytoplasmic stressosomes (4–6). Each stressosome is a megadalton-scale complex of 60–80 proteins (7, 8) and is thought to be the stress-sensing component of the GSR. The putative sensor proteins in the stressosome are called RsbR proteins. Forty RsbR proteins are assembled on a scaffold of 20 RsbS proteins as non-heme globin dimers with their N-terminal globin domains facing out into the cytoplasm (8, 9). In an unstressed state, this 60-protein core complex can sequester up to 20 RsbT proteins. When the RsbR proteins are exposed to a stressor, the sequestered RsbT is released by the stressosome into the cytoplasm (10), where it can then activate the downstream signaling steps that culminate in σ^B^ activation (11–16). *B. subtilis* encodes at least five RsbR paralogs, termed RsbRA, RsbRB, RsbRC, RsbRD, and YtvA (17–19). YtvA is different from the other RsbR proteins in that, in place of an N-terminal globin domain, it bears a light-oxygen-voltage domain, and unlike the other paralogs, it is known to sense blue light (18, 19).

Why *B. subtilis* encodes four RsbR paralogs has been an enduring mystery, and until recently they were thought to be largely redundant (6, 20). One attractive hypothesis was that each paralog was responsible for sensing a different stressor, but recent evidence has refuted this notion. Our previous work with strains encoding each of the RsbR paralogs individually revealed that all four RsbR paralogs can respond to ethanol stress but that each paralog differs with respect to the dynamics of the σ^B^ response (21). More recent work challenging single-RsbR strains with multiple different stressors showed that RsbRA-only and wild-type (i.e., containing all four RsbR paralogs) strains always show a rapid and transient σ^B^ response, whereas strains containing RsbRB, RsbRC, or RsbRD alone show different response profiles depending on the stressor (22). Thus, one apparent function of the different RsbR homologs is to modulate the intensity and duration of the consequent σ^B^ response when *B. subtilis* cells encounter an environmental stressor. It remains unknown how the RsbR homologs may impact σ^B^ responses in natural environments, as wild-type cells containing all four paralogs respond in a stereotypical way (with a rapid, transient σ^B^ response) under our laboratory conditions (21, 22). Nonetheless, our results with single-RsbR strains have shown in principle how the dynamics of the σ^B^ response can be modulated by stressosome proteins.

As the principal difference among strains encoding different individual RsbR paralogs appears to be the σ^B^ response profile (21, 22), we asked whether differences in σ^B^ response profile correspond with any functional consequences for the cell. As σ^B^ is part of a stress-response system, we reasoned that one important consequence might be the relative fitness—that is, the growth rate—of strains that are exposed to identical environmental stressors. Here, we compete wild-type and single-RsbR strains in pairwise co-cultures in the presence of environmental stressors. We find that wild-type and single-RsbR strains exhibit different competitive indices in different stressors, such that the most fit strain in one stressor is the least fit in another. Our data collectively suggest that the relative fitness of a strain under our experimental conditions is impacted by the dynamics of its σ^B^ response profile.

## RESULTS

### A differential fluorescence assay to measure competitive advantage

**Fig 1:**
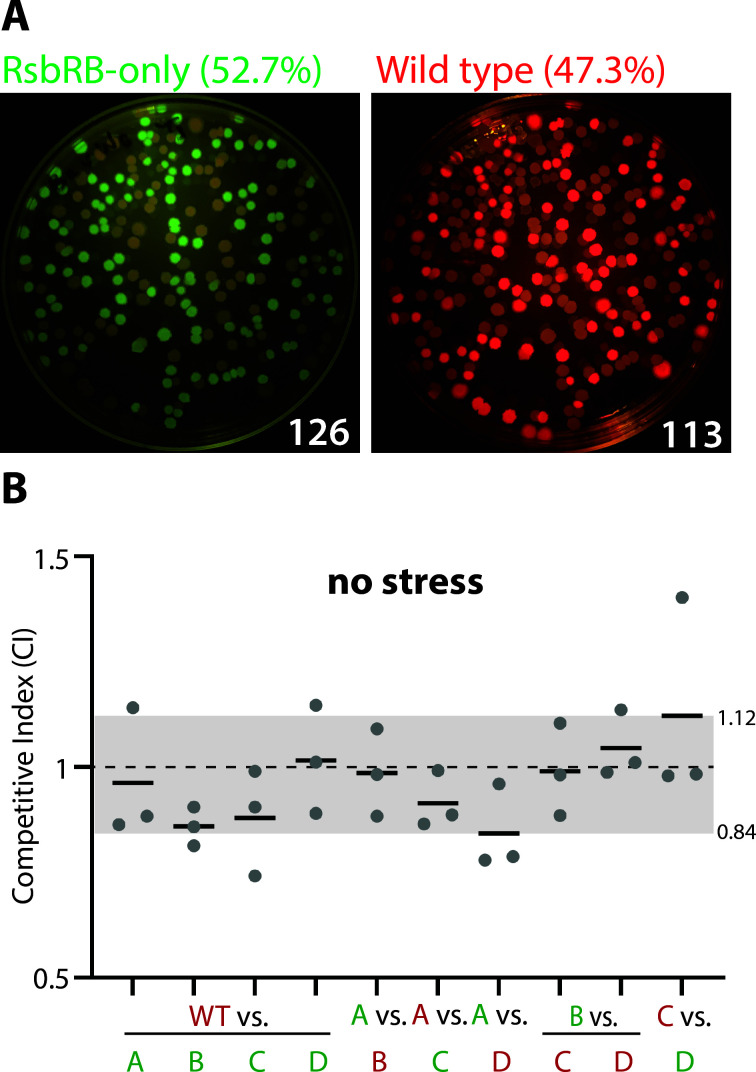
Relative enumeration and measurement error in co-cultured *B. subtilis* strains. (A) Differential fluorescence illumination of an agar plate spread with a dilution of a co-culture comprising a RsbRB-only strain labeled with GFP and a wild-type strain labeled with mKate2. The numbers of green- and red-fluorescent colonies are shown at the bottom right of each image. (**B)** Graph of competitive indices (CIs) of the listed strain pairs after 9 h in stress-free exponential-phase co-culture. Each gray dot represents one experiment, with the horizontal bars showing the mean CI of the experiments for each pair. The fluorescent label on each strain is indicated by the color of the X-axis label. The CI of each pair is calculated such that the top-listed strain is at an advantage if the CI is greater than 1. The gray shading shows the range of mean CIs for all the pairwise competitions. CI is calculated as (strain 1/strain 2 @ *t* = 9 h) / (strain 1/strain 2 @ *t* = 0). A, B, C, and D indicate RsbRA-only, RsbRB-only, RsbRC-only, and RsbRD-only strains. Green (GFP)-labeled WT (Δ*ytvA*), A, B, C, and D strains are CSS414, 415, 416, 417, and 418, respectively. Red (mKate2)-labeled WT (Δ*ytvA*), A, B, C, and D strains are CSS408, 409, 410, 411, and 412, respectively.

Given that strains containing single RsbR proteins showed different response profiles to an identical stressor (21), we asked whether different single-RsbR strains might show different fitness when challenged with identical environmental stressors. All of the strains we used were deleted for the RsbR-like blue-light sensor YtvA (18, 19) to eliminate any spurious σ^B^ activation by environmental light. To determine relative fitness, we used competitive co-cultures of strain pairs growing in stressor-containing media. In each co-culture, we inoculated approximately equal numbers of each strain (determined by optical density) into a stressor-containing medium and then sampled the relative strain populations initially (just after stressor addition) or after 9 h of growth. We maintained the co-cultures in the exponential phase (defined here as OD_600_ <0.6) via dilution with prewarmed fresh (stressor-containing) medium for two reasons. First, we sought to circumvent any physiological changes that could affect fitness due to cells entering the stationary phase. Second, we had gathered our σ^B^ response profile data from cells growing in exponential phase in microfluidic devices (21, 22), and we sought to examine the competitive index under similar conditions. To distinguish the co-cultured strains from one another, we labeled one or both strains with cytoplasmic green (GFP) or red (mKate2) fluorescent proteins under the control of a constitutive promoter (P*_hyperspank_*). We plated dilutions of the co-culture on solid media and enumerated the colony-forming units (CFU) with each fluorescent color in photographs taken under fluorescence illumination (Fig. 1A) (23). We elected to use a CFU-based method rather than other potential methods, such as relative DNA abundance, flow cytometry, or fluorescence microscopy, because of the simplicity and stringency of CFU counts, which exclusively consider the living cells of each strain.

### Single-RsbR strains display no inherent competitive advantage

Under non-stress conditions, σ^B^ is inactive, making it unlikely that the cellular complement of RsbR proteins would impact fitness in the absence of a stressor. To test this hypothesis, we co-cultured single-RsbR strains in exponential phase, in the absence of stress, for 9 h. We then calculated the competitive index (CI) of each co-culture as (A′/B′)/(A/B), where A and B are the proportions of strain 1 and 2, respectively, at *t* = 0, and A’ and B’ are the proportions of strain 1 and 2, respectively, at *t* = 9. Hence, a CI >1 indicates that strain 1 is outgrowing strain 2, whereas a CI <1 indicates that strain 1 is being outcompeted by strain 2. In the absence of stress and after 9 h of co-culture, the individual single-RsbR strain competitions showed slight variability, and the mean CI values for all the possible strain pairs ranged between 0.84 and 1.12, a range centered very close to 1.0 (Fig. 1B). These data imply that the single-RsbR strains we used do not have any inherent fitness advantage or disadvantage over one another. We also tested the effect of the different fluorescent labels on fitness by competing WT strains with each label against each other in the absence of stress or in 4% ethanol or 1 M NaCl. While the values ranged more widely, there was no consistent trend (Fig. S1), suggesting that the fluorescent label does not substantially affect fitness. These data also provide a reasonable estimate of the error in our experimental setup. Hence, we used the CI interval of 0.84 to 1.12 as a visual aid in figures to more easily observe CI values outside this range.

### Competition in 2% ethanol reveals no salient fitness differences

**Fig 2:**
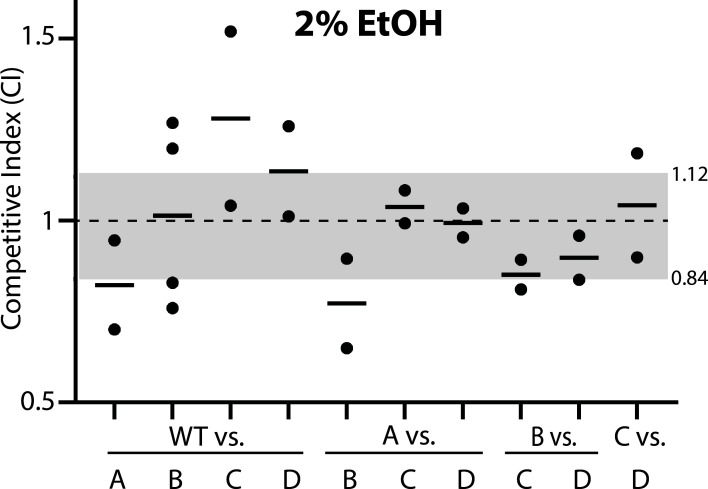
Competition among strain pairs co-cultured in the presence of 2% ethanol. The competitive indices of the listed strain pairs (A, RsbRA-only; B, RsbRB-only; C, RsbRC-only; D, RsbRD-only; WT, wild type (Δ*ytvA*) with all 4 RsbR paralogs) after 9 h of exponential-phase co-culture under 2% ethanol stress. Each black dot represents one experiment, with the horizontal bars showing the mean CI of the experiments for each pair. The gray shading shows the range of mean CIs for the stress-free control co-cultures (Fig. 1). At least one experiment was performed with each fluorescent label combination. Green (GFP)-labeled WT (Δ*ytvA*), A, B, C, and D strains are CSS414, 415, 416, 417, and 418, respectively. Red (mKate2)-labeled WT (Δ*ytvA*), A, B, C, and D strains are CSS408, 409, 410, 411, and 412, respectively.

Because we had previously detected different σ^B^ activation kinetics in different single-RsbR strains challenged with 2% ethanol (21, 22), we first co-cultured strain pairs in Luria-Bertani(LB) medium containing 2% ethanol to learn whether this stress level would reveal any fitness differences among single-RsbR strains. We plated cells just before stressor addition and maintained the co-cultures in exponential phase under stress conditions for 9 h before again plating to assess the relative fraction of each strain. Under these conditions, the majority of the co-cultures showed a minimal shift in their relative populations, with only a few experiments resulting in a mean CI outside the no-stress interval (Fig. 2). We interpreted these results as indicating that the 2% ethanol stressor was insufficient to uncover any substantial fitness differences, at least under the experimental conditions and 9-h interval we used.

### Competition in 4% ethanol reveals fitness led by RsbRA-only cells and trailed by RsbRD-only cells

**Fig 3:**
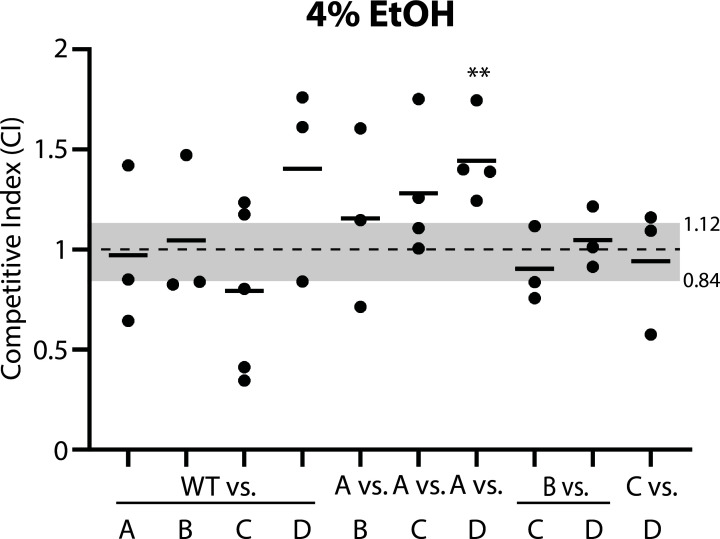
Competition among strain pairs co-cultured in the presence of 4% ethanol. The competitive indices of the listed strain pairs (A, RsbRA-only; B, RsbRB-only; C, RsbRC-only; D, RsbRD-only; WT, wild type (Δ*ytvA*) with all 4 RsbR paralogs) after 9 h of exponential-phase co-culture under 4% ethanol stress. Each black dot represents one experiment, with the horizontal bars showing the mean CI of the experiments for each pair. The gray shading shows the range of mean CIs for the stress-free control co-cultures (Fig. 1). At least one experiment was performed with each fluorescent label combination. **, *P* < 0.01 vs the same strain pair under non-stress conditions, unpaired 2-tailed *t*-test assuming equal variance. All other competitions were not significantly different from their non-stress counterparts. Green (GFP)-labeled WT (Δ*ytvA*), A, B, C, and D strains are CSS414, 415, 416, 417, and 418, respectively. Red (mKate2)-labeled WT (Δ*ytvA*), A, B, C, and D strains are CSS408, 409, 410, 411, and 412, respectively.

Our results with 2% ethanol prompted us to ask whether doubling the concentration of ethanol stressor would uncover fitness differences among single-RsbR strains. In 4% ethanol, a few clearer trends began to emerge. The first trend was that the RsbRA-only strain generally outcompeted every co-cultured strain except the wild-type, although only the competition with RsbRD-only cells reached statistical significance (Fig. 3). In contrast, the RsbRD-only strain generally lost against every other competitor except RsbRC-only cells (Fig. 3), placing it at the bottom of the fitness hierarchy. Cells containing only RsbRB or RsbRC were in the middle of the hierarchy, generally losing to RsbRA-only cells and winning against RsbRD-only cells but not reaching statistical significance (Fig. 3). Moreover, RsbRB-only and RsbRC-only cells showed no clear trend when co-cultured together (Fig. 3), indicating roughly equal fitness between these two strains. At the top of the fitness hierarchy, RsbRA-only cells and wild-type cells showed similar fitness, with mixed competition results among trials when these two strains were co-cultured (Fig. 3). As σ^B^ activation kinetics are presumably the primary difference between cells containing different RsbR paralogs, it is possible that the σ^B^ response pattern of a given strain influences its fitness in a specific stressful condition. Consistent with this idea, the strain pairs that showed roughly equal fitness (RsbRA-only/wild-type and RsbRB/RsbRC) in ethanol also show similar σ^B^ activation kinetics in ethanol (21, 22). Collectively, these experiments uncovered, as their strongest result, a superiority of RsbRA-only cells relative to RsbRD-only cells in 4% ethanol stress.

### Competition in 1 M NaCl shows fitness led by RsbRD-only cells and trailed by wild-type and RsbRA-only cells

**Fig 4:**
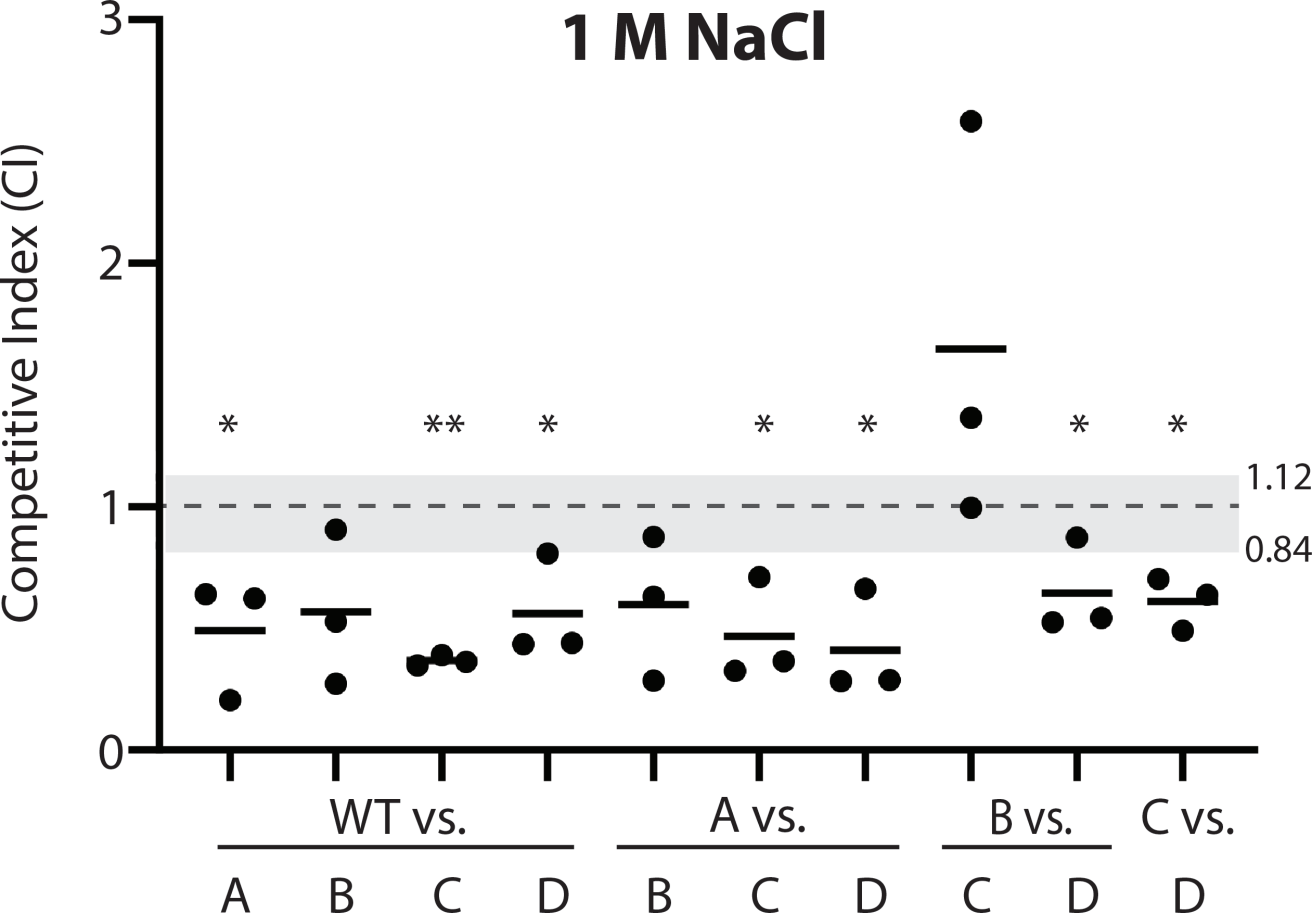
Competition among strain pairs co-cultured in the presence of 1 M NaCl. The competitive indices of the listed strain pairs (A, RsbRA-only; B, RsbRB-only; C, RsbRC-only; D, RsbRD-only; WT, wild type (Δ*ytvA*) with all 4 RsbR paralogs) after 9 h of exponential-phase co-culture under 1 M NaCl stress. Each black dot represents one experiment, with the horizontal bars showing the mean CI of the experiments for each pair. The gray shading shows the range of mean CIs for the stress-free control co-cultures (Fig. 1). At least one experiment was performed with each fluorescent label combination. *, *P* < 0.05, **, *P* < 0.01 vs the same strain pair under non-stress conditions, unpaired 2-tailed *t*-test assuming equal variance. All other competitions were not significantly different from their non-stress counterparts. Green (GFP)-labeled WT (Δ*ytvA*), A, B, C, and D strains are CSS414, 415, 416, 417, and 418, respectively. Red (mKate2)-labeled WT (Δ*ytvA*), A, B, C, and D strains are CSS408, 409, 410, 411, and 412, respectively.

Our observation of fitness differences among different single-RsbR strains in 4% ethanol made us ask whether the same differences would manifest in a different environmental stressor. We therefore competed with the same strain pairs in 1 M NaCl, which is also well-known to induce σ^B^ in *B. subtilis* (3). In NaCl, the fitness differences were typically more dramatic than in ethanol, with greater population shifts and more statistically significant differences (Fig. 4). Strikingly, the hierarchy that emerged in NaCl was nearly opposite what we observed in ethanol. The RsbRD-only strain, which was generally outcompeted in ethanol, was strongly dominant in NaCl, significantly outcompeting every other strain (Fig. 4). The RsbRA-only strain, in contrast, was outcompeted by every other single-RsbR strain and won only against wild-type cells, which lost every competition (Fig. 4). The RsbRB-only and RsbRC-only strains were again in the middle of the hierarchy, with RsbRB as the only strain to not significantly outcompete WT and RsbRA-only strains (Fig. 4). The contrasting results for co-cultures of RsbRA-only and RsbRD-only cells between 4% ethanol and 1M NaCl led us to investigate whether there were any obvious differences in cell chaining (24) that might explain the differences in colony-forming units when the co-cultures were plated. We observed minimal chaining under 4% ethanol stress (Fig. S2A), whereas cells under 1 M NaCl stress showed chaining and strikingly enlarged cell size (Fig. S2B). A limited analysis of chaining showed a slightly greater degree of chaining by RsbRD-only cells than RsbRA-only cells (Fig. S2B); however, such a trend would serve to underreport the number of RsbRD-only cells and so is insufficient to explain the observed greater proportion of RsbRD-only cells under 1 M NaCl stress (Fig. 4). These results suggest not only that the RsbR paralog(s) present in a strain influence its fitness under environmental stress, but also that a given genotype (and the consequent σ^B^ activation dynamics) may be beneficial or detrimental depending on the stressor encountered by the cells.

### Strains bearing hybrid RsbR proteins show an advantage for an RsbRA/B hybrid but a disadvantage for an RsbRD/A hybrid

**Fig 5:**
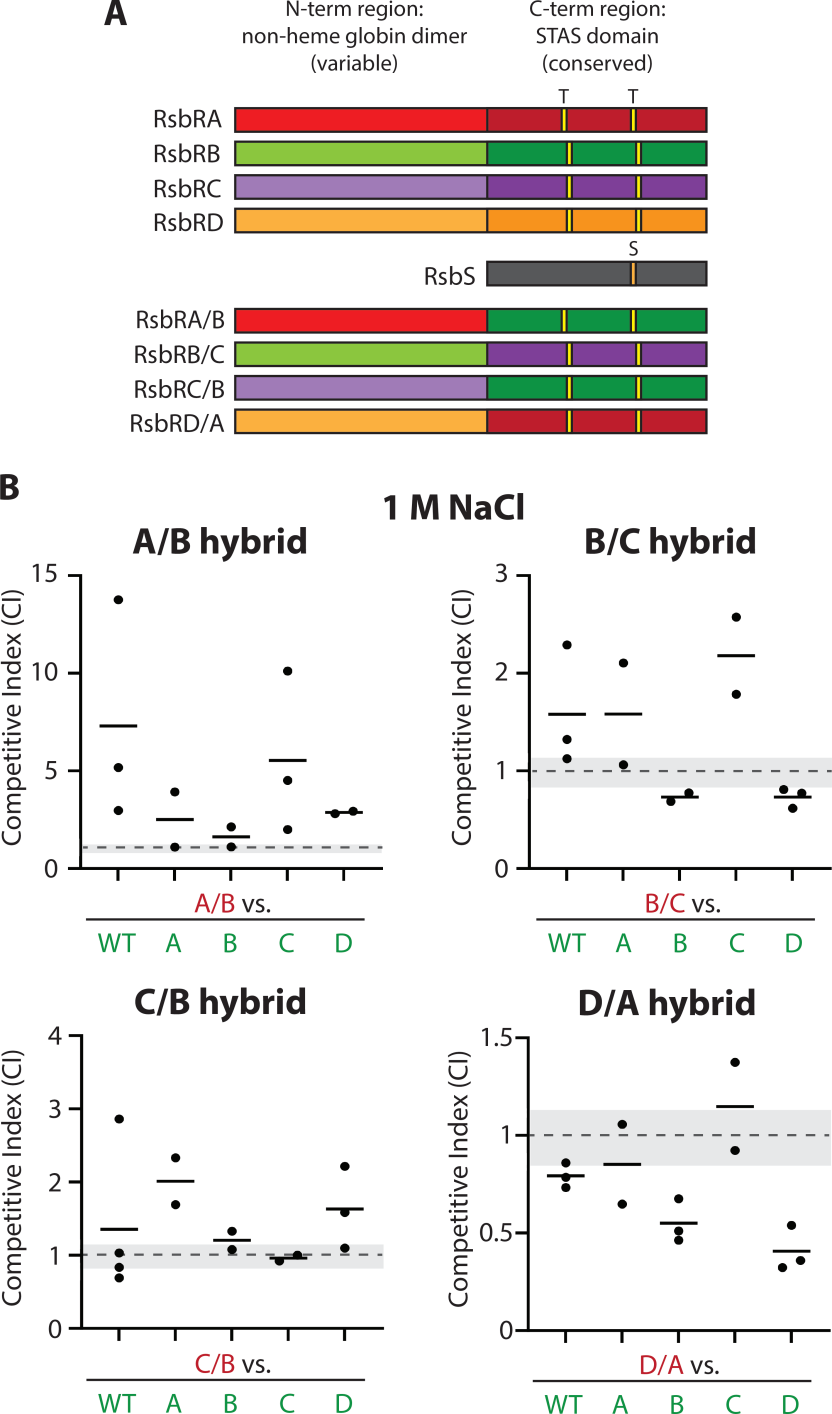
Competition among hybrid and single-RsbR strain pairs co-cultured in the presence of 1 M NaCl. (A) Schematic illustration showing each RsbR paralog and highlighting the N-terminal non-heme globin dimer region, which varies among the paralogs, and the more-conserved C-terminal STAS domain, which is homologous to the RsbS protein in the core of the stressosome. The hybrid RsbR paralogs were constructed by joining the N-terminal region of one RsbR to the C-terminal region of another, as in reference (22).** (B)** The competitive indices of the listed strain pairs (A/B, RsbRA/B hybrid-only; B/C, RsbRB/C hybrid-only; C/B, RsbRC/B hybrid-only; D/A, RsbRD/A hybrid-only; A, RsbRA-only; B, RsbRB-only; C, RsbRC-only; D, RsbRD-only; WT, wild type (Δ*ytvA*) with all 4 RsbR paralogs) after 9 h of exponential-phase co-culture under 1 M NaCl stress. Each black dot represents one experiment, with the horizontal bars showing the mean CI of the experiments for each pair. The gray shading shows the range of mean CIs for the stress-free control co-cultures (Fig. 1). Note the different Y scales for the different graphs. The fluorescent label on each strain is indicated by the color of the X-axis label. Green (GFP)-labeled WT (Δ*ytvA*), A, B, C, and D strains are CSS414, 415, 416, 417, and 418, respectively. Red (mKate2)-labeled A/B, B/C, C/B, and D/A strains are CSS1261, 1264, 1267, and 1258, respectively.

We took advantage of the clear fitness differences of single-RsbR strains in 1 M NaCl to make a preliminary examination of how strains bearing single, hybrid RsbR proteins would compare to wild-type or single-RsbR strains. We constructed the hybrid strains by creating gene fusions coupling the N-terminal half (globin domain) of one RsbR paralog to the C-terminal half (STAS domain) of another, as in our previous work (Fig. 5A) (22). Each hybrid gene was expressed as the only source of RsbR protein in the cell via allelic replacement at the locus of the C-terminal half (e.g., the *rsbRA/B* fusion was expressed from the native promoter at the *rsbRB* locus (22)). We deployed four different fusions (Fig. 5A), competing each with the wild-type and each single-RsbR strain in 1 M NaCl. We chose these specific fusions so that a segment of each of the four paralogs was represented at least once, so that there was at least one pair of fusions with the same C-terminal region but a different N-terminal region (A/B and C/B), and one reciprocal fusion (B/C and C/B). We did not perform no-stress controls with these 20 strain pairs, and hence significance cannot be calculated; these results must be considered as trends only. Nonetheless, and unexpectedly, the RsbRA/B hybrid strain outcompeted all the other strains, including the RsbRD-only strain (Fig. 5B). In contrast, the RsbRD/A hybrid strain was at the bottom of the hierarchy after generally being outcompeted by single-RsbR strains (Fig. 5B), despite having the N-terminal half of RsbRD. An RsbRB/C hybrid, consistent with its constituent parts deriving from strains with intermediate fitness, was in the middle of the hierarchy, losing to RsbRB- and RsbRD-only strains but outcompeting wild-type, RsbRA-only, and RsbRC-only strains (Fig. 5B). Interestingly, the RsbRC/B hybrid showed a different pattern from the RsbRB/C hybrid, generally winning against RsbRA-only and RsbRD-only strains but generally equal to the wild-type and RsbRB- and RsbRC-only strains (Fig. 5B). These results further illustrate the influence of RsbR protein identity and sequence on relative growth rate under stress conditions. For example, replacing the C-terminal half of RsbRD with the analogous sequence from RsbRA severely affected fitness in 1 M NaCl, causing cells to drop from the top of the fitness hierarchy to its bottom. This particular result suggests that the C-terminal portion of RsbRD is important for its enhanced fitness in 1 M NaCl; however, the overall amplitude of the σ^B^ response may also contribute. Consistent with this interpretation, while RsbRD-only cells show a strong σ^B^ response to NaCl, the RsbRD/A and RsbRB/C hybrids are characterized by a weak response, at least to 2% ethanol (22). A weaker response may make these hybrids less fit than RsbRD-only cells in 1 M NaCl, whereas the RsbRA/B and RsbRC/B hybrids, which have a stronger response in 2% ethanol (22), generally outcompeted RsbRD-only cells (Fig. 5B). Our hybrid results also suggest that fitness, at least in 1 M NaCl, is not fully optimized with the native protein sequences, as the RsbRA/B hybrid outcompeted all the native single-RsbR strains and the wild-type.

### The presence of RsbRA is detrimental to fitness in 1 M NaCl but is advantageous in 4% ethanol

The competition between single-RsbR strains in 1 M NaCl showed a clear fitness deficit for strains with RsbRA as the only source of RsbR in the cell, suggesting that the transient RsbRA-type σ^B^ response profile (21, 22) is detrimental to fitness under NaCl stress conditions. Consistent with this notion, wild-type cells, which have a transient response profile very similar to RsbRA-only cells (21, 22), were also outcompeted by every single-RsbR strain (Fig. 4). The similarity between RsbRA-only and wild-type cells with respect to their σ^B^ response profiles led us to speculate that RsbRA dominates the overall response, even when (as in the wild type) other RsbR paralogs are present (21). One prediction of this model is that deleting *rsbRA* from a wild-type strain would abrogate its transient σ^B^ response profile, converting it to a sustained-type response. Because the wild-type (and RsbRA-only) strains were the least fit under 1 M NaCl treatment (Fig. 4), we further hypothesized that deletion of *rsbRA* from a wild-type strain would increase its fitness in NaCl. Conversely, because wild-type cells were at the top of the fitness hierarchy in 4% ethanol, we reasoned that *rsbRA* deletion might compromise fitness under ethanol stress.

**Fig 6:**
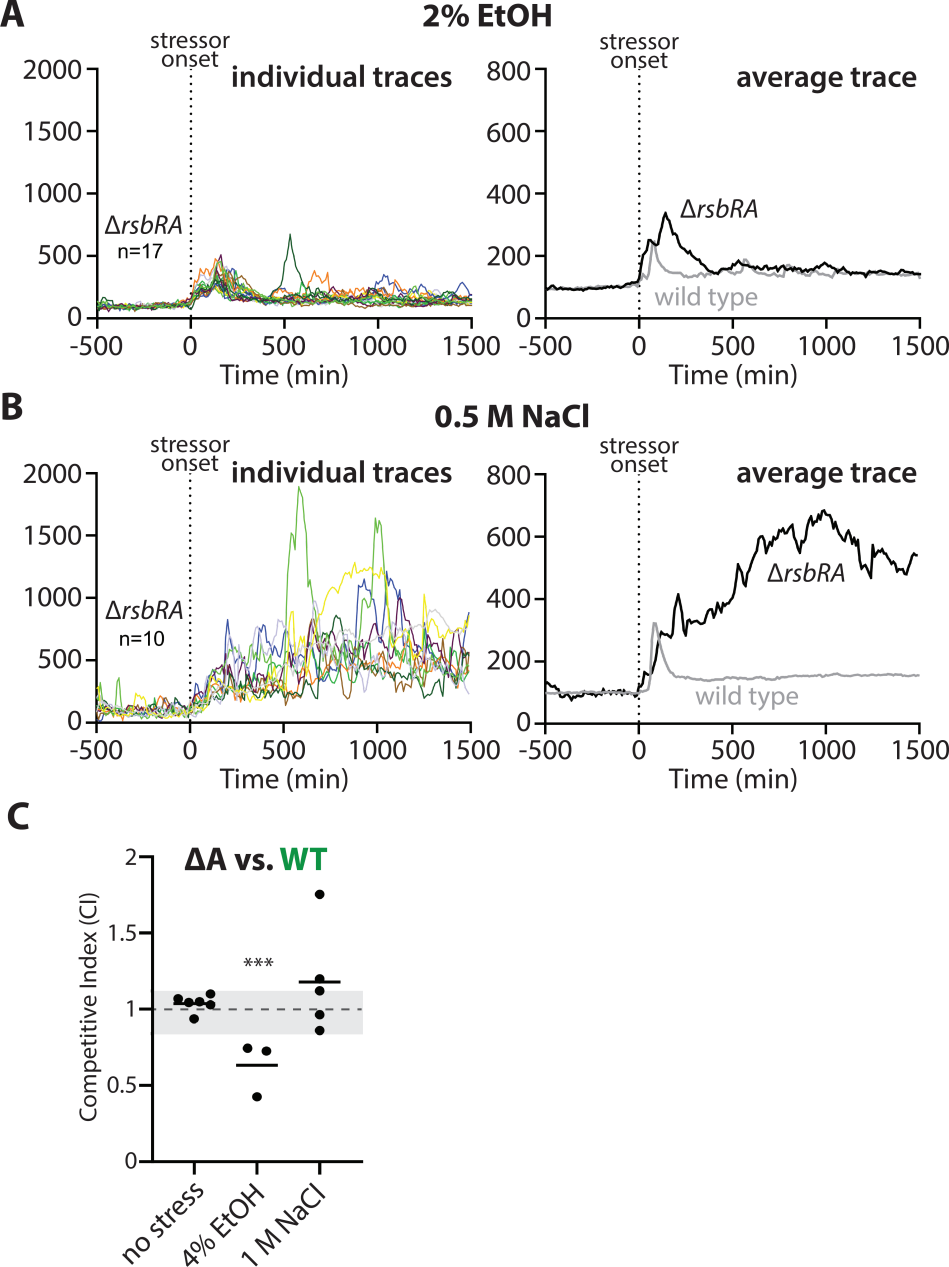
Impact of absent RsbRA on σ^B^ response profiles and fitness in 4% ethanol and 1 M NaCl. (A, B) Reporter traces of strains bearing a P*_rsbV_*-mNeonGreen (σ^B^-responsive) fluorescent transcriptional reporter and exposed to stress in a microfluidic device. (**A)** Left panel, individual lineage traces of a Δ*rsbRA* strain (MTC1973) exposed to 2% ethanol at the zero time point. Right panel, the average trace (black) compared with the average trace of wild-type cells exposed to 2% ethanol [gray; MTC1801 data taken from reference (22)].** (B)** Left panel, individual lineage traces of a Δ*rsbRA* strain exposed to 0.5 M NaCl at the zero time point. right panel, the average trace (black) compared with the average trace of wild-type cells exposed to 0.5 M NaCl [gray; MTC1801 taken from reference (22)].** (C)** The competitive indices of the listed strain pairs (ΔA, *ΔytvA* Δ*rsbRA*, MTC1683; WT, wild type (Δ*ytvA*) with all 4 RsbR paralogs, GFP-tagged, CSS414) after 9 h of exponential-phase co-culture under no stress, 4% ethanol stress, or 1 M NaCl stress as indicated. Each black dot represents one experiment, with the horizontal bars showing the mean CI of the experiments for each pair. The gray shading shows the range of mean CIs for the stress-free control co-cultures (Fig. 1). ***, *P* < 0.001 vs the same co-culture under non-stress conditions, unpaired 2-tailed *t*-test assuming equal variance.

We first tested our prediction about how *rsbRA* deletion would impact σ^B^ profiles. We used a strain deleted only for *rsbRA* (and *ytvA* to prevent activation by environmental light or fluorescence microscopy) and bearing a σ^B^-responsive mNeonGreen transcriptional reporter. We then monitored the σ^B^ activity of cells before and after stressor addition in a microfluidic device, as in our previous work (21, 22). We compared the σ^B^ profiles of the Δ*rsbRA* strain exposed to 2% ethanol or 500 mM NaCl to data we previously obtained for wild-type cells under the same stressor conditions (22). We used these concentrations, which are half the concentrations used for the competition experiments, for consistency with our previous data from microfluidics experiments (22). As we predicted, deletion of *rsbRA* impacted the σ^B^ response profile, eliminating the sharp response peak of the wild-type strain and replacing it with a broader peak in ethanol or a sustained response in NaCl (Fig. 6A through B). Having established that the σ^B^ response profile is altered by deletion of *rsbRA*, we then tested our hypothesis that loss of RsbRA would abet fitness in NaCl but hamper fitness in ethanol. We competed the Δ*rsbRA* strain against the wild type in either 4% ethanol or 1 M NaCl, first co-culturing them in the absence of stress to establish that neither strain had an inherent advantage. In the absence of stress, neither strain had an advantage (Fig. 6C), whereas in 4% ethanol, the wild type significantly outcompeted the Δ*rsbRA* (Fig. 6C), consistent with RsbRA having a positive effect on fitness in ethanol. In contrast, in 1 M NaCl, the Δ*rsbRA* strain generally outcompeted the wild type (Fig. 6C). Although the result in NaCl was not statistically significant, it is at least in accord with RsbRA having an overall negative effect on fitness in NaCl. Overall, these experiments suggest that a transient, wild-type-like response is a greater asset in ethanol than it is a liability in NaCl.

### Fitness differences are at least partially dependent on σ^B^

**Fig 7:**
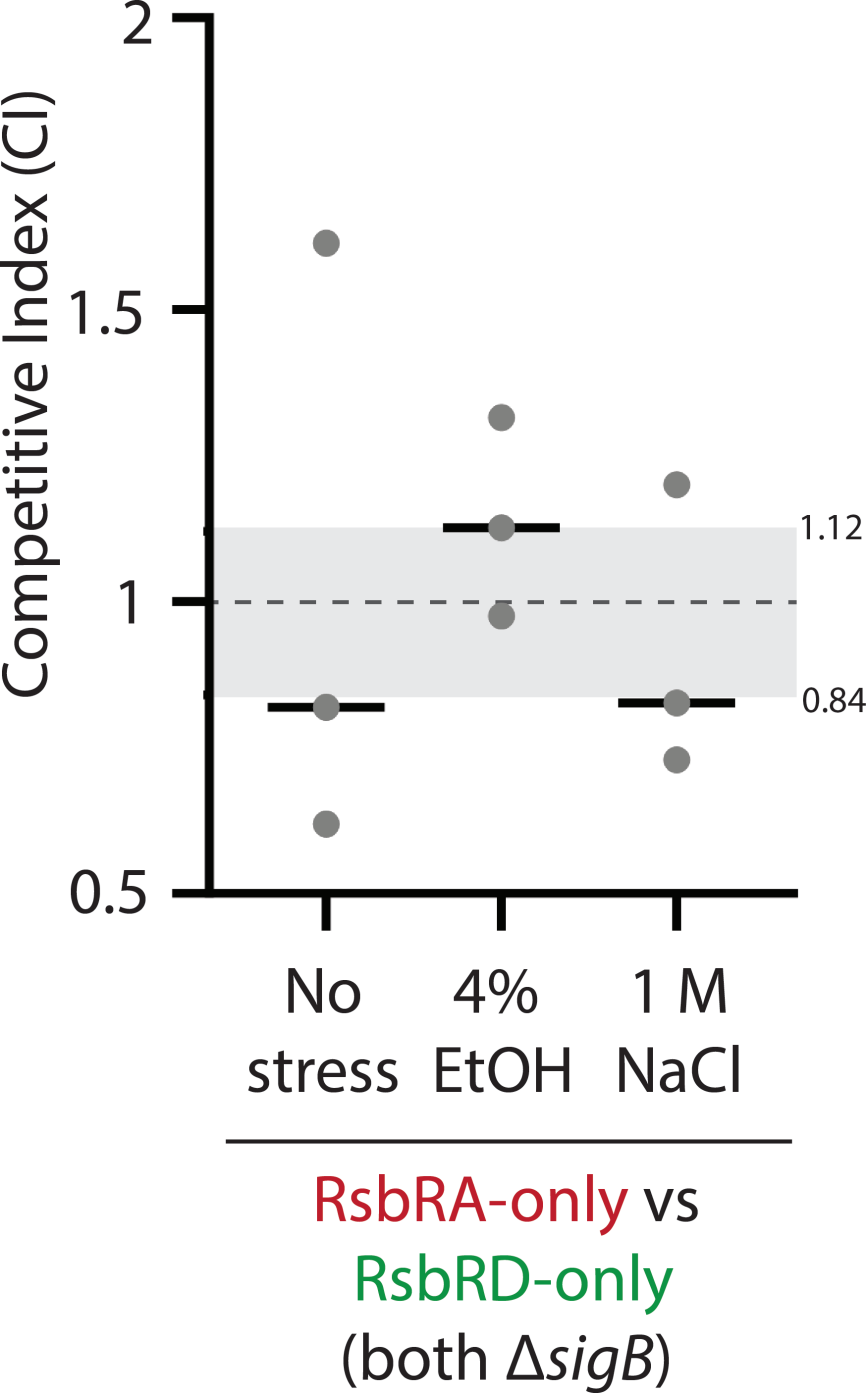
Role of σ^B^ in competitive outcomes under stress conditions. The competitive indices of the listed strain pairs after 9 h of exponential-phase co-culture under no stress, 4% ethanol stress, or 1 M NaCl stress are as indicated. The fluorescent label on each strain is indicated by the color of the X-axis label. Each dot represents one experiment, with the horizontal bars showing the mean CI of the experiments for each pair. The gray shading shows the range of mean CIs for the stress-free control co-cultures (Fig. 1). Neither of the RA-only vs RD-only competitions was significantly different from the no-stress control. RsbRA-only Δ*sigB*, mKate2 tagged, CSS2066; RsbRD-only Δ*sigB*, GFP tagged, CSS2078.

Because different RsbR proteins mediate different σ^B^ response profiles and show different fitness in competition stress co-cultures, we sought to formally test this connection by competing selected strains with *sigB* deletions, thus abolishing the σ^B^ response. If observed fitness differences depend on σ^B^ response profiles, then *sigB* deletion should eliminate such differences. We chose RsbRA-only and RsbRD-only strains for our test, as this strain pair showed strong, consistent, significant, and opposite fitness differences in 4% ethanol and 1 M NaCl (Fig. 3 and 4). Deletion of *sigB* from each strain in this strain pair resulted in smaller, non-significant deviations from the no-stress control (Fig. 7), suggesting that different σ^B^ responses indeed impact relative fitness. However, the mean CI values trended in the same direction as the *sigB*-replete competitions (Fig. 7), raising the intriguing possibility that RsbR proteins might also impact fitness via σ^B^-independent pathways.

## DISCUSSION

Manipulation of the complement of proteins present in the stressosome can clearly impact the dynamics—the magnitude and duration—of the *B. subtilis* σ^B^ response (21, 22). Our results here provide evidence that different σ^B^ response dynamics not only affect cell fitness but also that the benefit or detriment of a given response pattern varies depending on the stressor. We found that the characteristic wild-type σ^B^ response, characterized by its transience, appears to benefit cells under ethanol stress but is at a relative disadvantage under NaCl stress (Fig. 4). Conversely, a sustained (i.e., repeated in single cells) response confers an advantage under NaCl stress but a disadvantage under ethanol stress. Our experiments competing hybrid RsbR-bearing strains with single-RsbR strains (Fig. 5) and competing the wild type with the Δ*rsbRA* strain (Fig. 6) suggest that the σ^B^ response profile, as influenced by the particular RsbR proteins in the stressosome, is primarily responsible for the relative fitness of a strain under environmental stress. This conclusion is further bolstered by our results showing that ∆*sigB* strain pairs no longer show significantly different fitness (Fig. 7).

One implication of this finding is that the optimally fit σ^B^ response to one stressor may be different from the optimal response to a different stressor. Under our laboratory conditions, wild-type cells do not appear to change their σ^B^ profiles in response to different stressors (22). Hence, one outstanding question is whether *B. subtilis* cells in natural environments modulate their σ^B^ responses to optimize fitness under particular conditions. There are many control points that cells may deploy, such as the expression and protein levels of different RsbR paralogs, which may even change over time or in response to stress in ways that have not yet been measured but that may impact fitness or survival. Another important question for the future is how a particular σ^B^ response profile impacts fitness when cells face simultaneous or sequential stresses. How would RsbRA-only and RsbRD-only cells compare if exposed to *both* ethanol and NaCl stress?

Another interesting and non-exclusive possibility raised by our work is that wild-type cells may not be optimized for all stressors. In fact, wild-type cells were among the least fit (only the RsbRD/A hybrid was less fit) of all the tested strains under NaCl stress under our experimental conditions. Perhaps cells are forced into a trade-off, such that wild-type cells trade maximal fitness under certain stress conditions in favor of increased fitness across a broad range of stressors. That a trade-off exists is at least supported by our data showing that a strain with the greatest fitness under one condition (RsbRD-only cells in NaCl, for example, Fig. 4) was the least fit under another condition (in ethanol, Fig. 3).

We do not yet understand why particular σ^B^ response profiles yield the observed fitness advantages or disadvantages of different stressors. One salient pattern that emerged is that the wild-type/RsbRA-only σ^B^ profile, characterized by its transience and synchrony (21, 22), is associated with greater fitness in ethanol and poorer fitness in NaCl. As other single-RsbR strains typically show repeated responses in individual cells that manifest as a sustained response when averaged across many cells (21, 22), it is unclear why RsbRD-only cells show a consistent fitness advantage in NaCl. The different fitness results we observed for the hybrid-RsbR strains suggest that subtle differences in σ^B^ response profiles may influence fitness in ways that we will only understand after further study of how hybrid-RsbR strains respond to different stressors.

Finally, we return to the question of why *B. subtilis* encodes four RsbR paralogs. At first glance, our results appear to support the idea that they are, in wild-type cells at least, largely redundant, as wild-type cells respond in a stereotyped way to multiple stressors (22). However, our genetic manipulations that force cells to use just one paralog highlight the flexibility and breadth of responses that are possible using the stressosome-based GSR pathway of this organism. It is thus tempting to speculate that the different responses and fitness that we observe in genetically manipulated, laboratory-grown, exponential-phase cells in rich medium reflect flexibility that exists in nature but that is difficult to detect in the laboratory. Indeed, the σ^B^ protein itself appears much more important for fitness and survival under extreme stress or stationary-phase conditions than in the exponential phase (25–27). For example, in slow-growing or stationary-phase cells exposed to environmental stressors, perhaps the differential stability of different RsbR proteins becomes a factor. Alternatively, stressosome composition might be altered on long timescales in cells that become adapted to different stressors.

## MATERIALS AND METHODS

### Strains and growth conditions

The bacterial strains used in this study are listed in Table 1. *B. subtilis* strains were grown in LB Lennox broth (10 g/L tryptone, 5 g/L yeast extract, and 5 g/L NaCl) or on Lennox agar plates fortified with 1.5% Bacto agar at 37°C. As LB already contains NaCl, strains being prepared for NaCl stress were grown in salt-free LBK medium (10 g/L tryptone, 5 g/L yeast extract, 21 mM K_2_HPO_4_, 11 mM KH_2_PO_4_), as in our previous work (22). When appropriate, antibiotics were added (5 µg/mL chloramphenicol or 100 µg/mL spectinomycin) to select for markers. Markerless replacement of *rsbR* genes with hybrid versions was performed using the pMiniMAD vector for allelic replacement. Details of strain construction are given in Supplemental Text S1.

**TABLE 1:** Strains used in this study

Strain	Relevant genotype or description	Source or reference
CSS408	3610 Δ*ytvA amyE*::P_hyperspank_-mKate2 (Cm^R^) (WT)	This study
CSS409	3610 Δ*ytvA* Δ*rsbRB* Δ*rsbRC* Δ*rsbRD amyE*::P_hyperspank_-mKate2 (Cm^R^) (RsbRA-only)	This study
CSS410	3610 Δ*ytvA* Δ*rsbRA* Δ*rsbRC* Δ*rsbRD amyE*::P_hyperspank_-mKate2 (Cm^R^) (RsbRB-only)	This study
CSS411	3610 Δ*ytvA* Δ*rsbRA* Δ*rsbRB* Δ*rsbRD amyE*::P_hyperspank_-mKate2 (Cm^R^) (RsbRC-only)	This study
CSS412	3610 Δ*ytvA* Δ*rsbRA* Δ*rsbRB* Δ*rsbRC amyE*::P_hyperspank_-mKate2 (Cm^R^) (RsbRD-only)	This study
CSS414	3610 Δ*ytvA amyE*::P_hyperspank_-GFP (Cm^R^) (WT)	This study
CSS415	3610 Δ*ytvA* Δ*rsbRB* Δ*rsbRC* Δ*rsbRD amyE*::P_hyperspank_ -GFP (Cm^R^) (RsbRA-only)	This study
CSS416	3610 Δ*ytvA* Δ*rsbRA* Δ*rsbRC* Δ*rsbRD amyE*::P_hyperspank_ -GFP (Cm^R^) (RsbRB-only)	This study
CSS417	3610 Δ*ytvA* Δ*rsbRA* Δ*rsbRB* Δ*rsbRD amyE*::P_hyperspank_ -GFP (Cm^R^) (RsbRC-only)	This study
CSS418	3610 Δ*ytvA* Δ*rsbRA* Δ*rsbRB* Δ*rsbRC amyE*::P_hyperspank_ -GFP (Cm^R^) (RsbRD-only)	This study
CSS1258	3610 Δ*ytvA* Δ*rsbRB* Δ*rsbRC* Δ*rsbRD rsbRA::rsbRD/A amyE*::P_hyperspank_-mKate2 (Cm^R^) RsbRD/A hybrid as the only source of RsbR within the cell	This study
CSS1261	3610 Δ*ytvA* Δ*rsbRA* Δ*rsbRC* Δ*rsbRD rsbRB::rsbRA/B amyE*::P_hyperspank_-mKate2 (Cm^R^) RsbRA/B hybrid as the only source of RsbR within the cell	This study
CSS1264	3610 Δ*ytvA* Δ*rsbRA* Δ*rsbRB* Δ*rsbRD rsbRC::rsbRB/C amyE*::P_hyperspank_-mKate2 (Cm^R^) RsbRB/C hybrid as the only source of RsbR within the cell	This study
CSS1267	3610 Δ*ytvA* Δ*rsbRB* Δ*rsbRC* Δ*rsbRD rsbRB::rsbRC/B amyE*::P_hyperspank_-mKate2 (Cm^R^) RsbRC/B hybrid as the only source of RsbR within the cell	This study
CSS2066	3610 Δ*sigB* Δ*ytvA* Δ*rsbRB* Δ*rsbRC* Δ*rsbRD amyE*::P_hyperspank_-mKate2 (Cm^R^) (RsbRA-only, no σ^B^)	This study
CSS2078	3610 Δ*sigB* Δ*ytvA* Δ*rsbRA* Δ*rsbRB* Δ*rsbRC amyE*::P_hyperspank_-GFP (Cm^R^) (RsbRD-only, no σ^B^)	This study
MTC 1683	3610 Δ*ytvA* Δ*rsbRA*; stressosome containing all RsbR paralogs except RsbRA	This study
MTC 1973	3610 *hag*_A223V_ Δ*ytvA* Δ*rsbRA amyE*::*DG364-*P_hyperspank_*-mNeptune* (Cm^R^) *ywrK*::*DG1730-P_rsbV_-mNeonGreen* (Spc^R^); stressosome containing all RsbR paralogs except RsbRA	This study

### Competition co-cultures and stress experiments

Strains were grown overnight, back-diluted at a 1:1,000 ratio in 3 mL of LB (for ethanol-stress experiments) or LBK (for NaCl-stress experiments), and then grown for 2–3 h to bring cells into exponential phase (OD_600_ <0.6). Using the OD_600_ of these starter cultures, an initial inoculum volume was calculated for each strain such that the initial OD_600_ of the co-culture would be approximately 0.005 for no-stress or ethanol-stressed cultures and 0.05 for NaCl-stressed cultures, with approximately equal numbers of cells for each strain. To distinguish between strains, either one (for Δ*rsbRA* experiments) or both strains (all other experiments) were fluorescently tagged with a constitutively produced GFP or red (mKate2) fluorescent protein. Both strains were then inoculated into a 25-mL flask of LB (for ethanol) or LBK (for NaCl) and agitated to mix. The stressor was added to its final concentration immediately thereafter (unless the competition was a no-stress control culture). Ethanol was added to its final concentration (2% or 4%) from a 100% ethanol stock to swirled LB cultures (to dissipate the concentrated stressor immediately). NaCl was added to swirled cultures at 1 M from a 2 M NaCl stock in LBK. Samples (100 µL) were taken and serially diluted for plating (on plain LB agar) immediately after stressor addition and then after 9 h of shaking culture (37°C, 180 rpm). The OD_600_ was monitored visually, and cultures were diluted when necessary (as they approached OD_600_ of 0.5; 3–5 times over 9 h) at a 1:100 ratio (NaCl) or 1:1,000 ratio (no stress, ethanol) with fresh, prewarmed, stressor-containing medium to ensure that cultures remained in exponential phase, defined here as OD_600_ <0.6. The plates were grown overnight at 37℃, images of the resulting CFU on plates with countable colony density were taken using appropriate fluorescence illumination and filters (RFP or GFP) (23), and colonies were counted using ImageJ (crosshairs tool). When multiple plates and/or serial dilutions yielded well-countable colonies for both strains, the colony numbers were averaged for the dilutions. The colony counts for each competition are given in online supplementary file 2. All competition trials were completed at least twice in separate experiments and at least three times for the assessment of statistical significance. The competitive index was calculated as (A′/B′)/(A/B), where A and B are the proportions of strains 1 and 2, respectively, at *t* = 0, and A’ and B’ are the proportions of strains 1 and 2, respectively, at *t* = 9. Significance was evaluated by 2-tailed *t*-testing assuming equal variance, comparing the CI values of a no-stress co-culture with its stressed counterpart.

### Microfluidic microscopy and data curation

Microfluidic experiments were performed essentially as described in our previous work (22). All microfluidic plumbing was designed and set up as in our previous work (22). The relevant media were LB and LB + 2% ethanol or LBK and LBK + 500 mM NaCl. Medium switching was performed as previously described (22).

### Automated imaging and lineage tracking

Light microscopy was performed on a Nikon Eclipse Ti inverted microscope equipped with a Photometrics Prime 95B sCMOS camera, a 100× Plan Apo oil objective (NA 1.45, Nikon), an automated stage (Nikon), a Lumencor SOLA SE II 365 Light Engine fluorescent illumination system, and an OKO temperature-controlled enclosure in which the temperature was maintained at approximately 37°C during imaging. Image acquisition was performed using NIS-Elements AR 5.11.03 64-bit. mNeonGreen (used for σ^B^ reporters) was imaged at approximately 33% illumination power with 200 ms exposures, and phase contrast was used for cell visualization. Images were captured at 10-min intervals. Cell lineages were manually curated, tracked, and plotted as in our previous work (22).

## SUPPLEMENTAL MATERIAL

Data S2 msphere.00719-23-s0001.xlsx Colony count data.

Text S1 and Supplemental Figures msphere.00719-23-s0002.pdf Fig. S1 and S2 and modes of strain construction.

ASM does not own the copyrights to Supplemental Material that may be linked to, or accessed through, an article. The authors have granted ASM a non-exclusive, world-wide license to publish the Supplemental Material files. Please contact the corresponding author directly for reuse.
