# Supplementary material for: Temporal σB stress-response profiles impact Bacillus subtilis fitness
Source: mSphere. 2024 Jan 18;9(2):e00719-23. doi: 10.1128/msphere.00719-23 (PMC10900884; doi:10.1128/msphere.00719-23)
Supplement: Text S1 and Supplemental Figures — Fig. S1 and S2 and modes of strain construction. [file msphere.00719-23-s0002.pdf]

Supplemental Figures

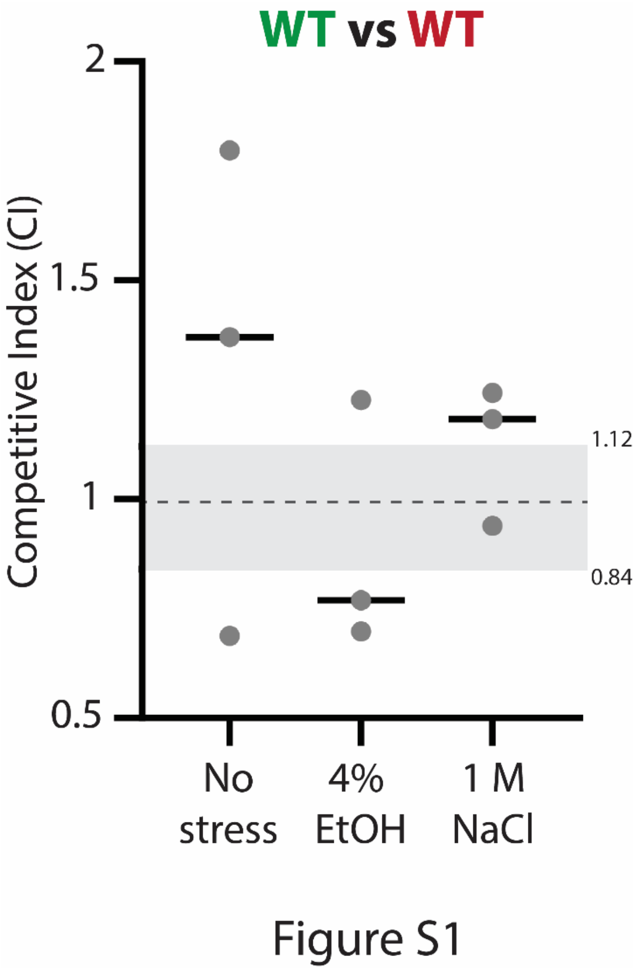

Figure S1. Co-cultures of wild-type strains with different constitutive fluorescent markers. Wild-type (Δ*ytvA*) strains carrying constitutive green fluorescent protein (GFP; CSS414) or red fluorescent protein (mKate2; CSS408) were co-cultured in the absence of stressor, with 4% ethanol, or with 1 M NaCl for 9 h in exponential phase before plating and relative CFU enumeration. Competitive index was calculated by comparison with the strain ratios at the time of stressor addition (t = 0). The shaded region denotes the range of CI values obtained for strain pairs cultured in the absence of stress (Fig. 1).

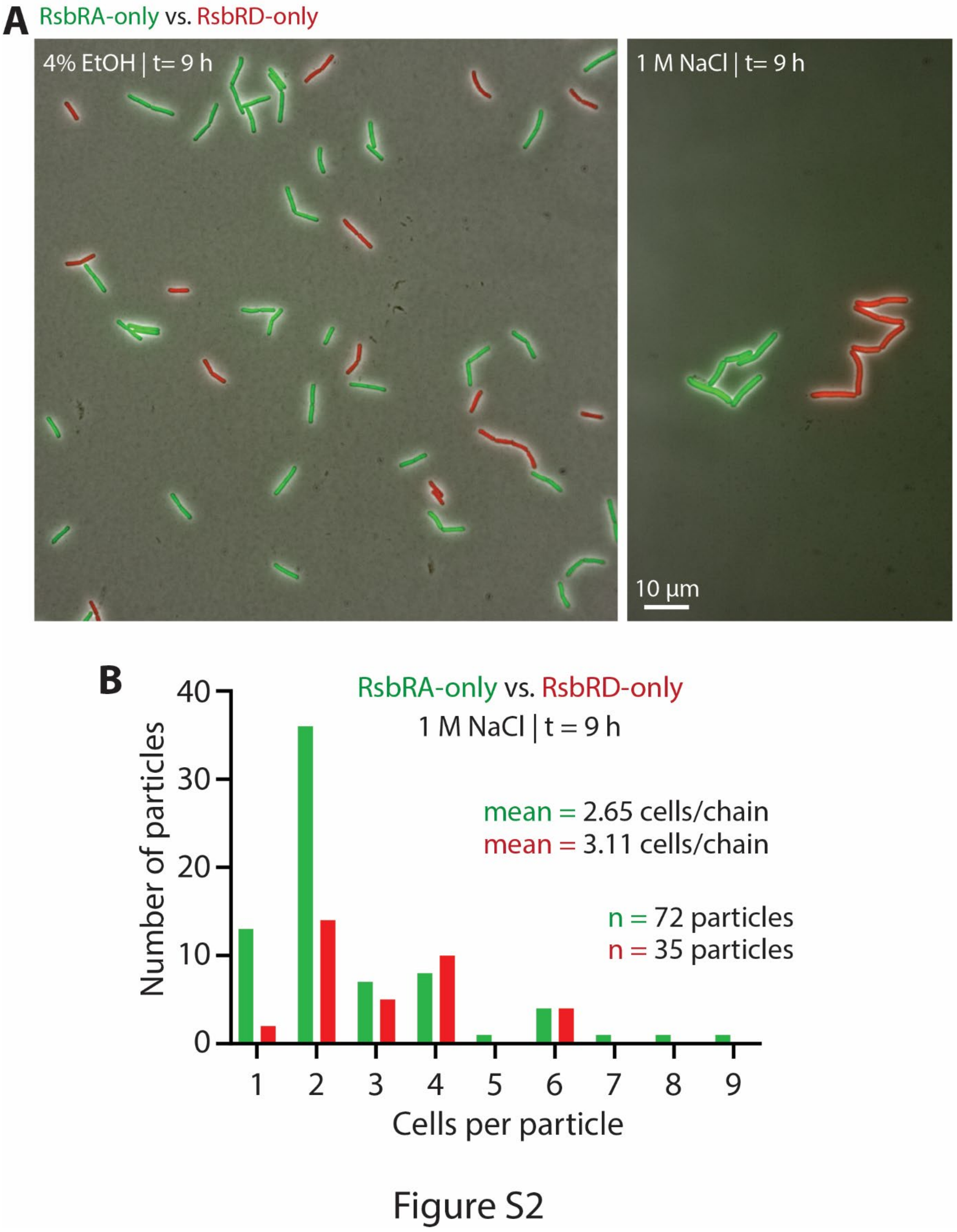

Figure S2. Analysis of cell chaining in co-cultures treated with ethanol or NaCl. An RsbRA-only strain carrying constitutive green fluorescent protein (GFP; CSS415) was co-cultured with an RsbRD-only strain carrying constitutive red fluorescent protein (mKate2; CSS412) in the presence of 4% ethanol

or with 1 M NaCl for 9 h in exponential phase before fluorescence imaging on agarose pads. **A.** Representative images of cells at t = 9 h of culture in ethanol or NaCl as noted. Both images are at the same scale. **B.** Histogram of cell chain length as manually determined in fields taken from the NaCl-treated sample; a "particle" is a unit of one or more connected cells.

Detailed Modes of Strain Construction

The markerless gene replacement in the strains below were added in succession using the pminiMAD vector (gift of Daniel Kearns). A pminiMAD vector propagated in E. coli and containing the desired gene was directly transformed into PY79 via competence and selected on MLS (0.5 µg/ml erythromycin and 2.5 µg/ml lincomycin). A phage SPP1 lysate was prepared from that intermediate strain, and the recipient strain was phage-transduced with the PY79 strain containing the desired chromosomally integrated pMiniMAD vector and again selected on MLS. Five to 10 transductants were then inoculated into liquid LB and kept in exponential phase at approximately 25°C for several hours to permit plasmid excision before being repeatedly diluted and grown in liquid LB at 37°C (restrictive for plasmid replication) to promote loss of excised plasmid. The cells were then plated, and single colonies were screened for the successful replacement by colony PCR, restreaked for single colonies, patched on plain LB and LB/MLS plates to verify plasmid loss, restreaked, verified by PCR and stored at -80°C. Hybrid strain construction was performed as specified below using PY79 lysates from Hamm et al. (1).

CSS408

MTC1672 was transduced with lysate from PY79 containing constitutive *amyE*::$P_{hyperspank}$-mKate2 as described above to produce 3610 *ΔytvA amyE*::$P_{hyperspank}$-mKate2

CSS409

MTC1697 was transduced with lysate from PY79 containing constitutive *amyE*::$P_{hyperspank}$-mKate2 as described above to produce 3610 *ΔytvA ΔrsbRB ΔrsbRC ΔrsbRD amyE*::$P_{hyperspank}$-mKate2

CSS410

MTC1695 was transduced with lysate from PY79 containing constitutive *amyE*::$P_{hyperspank}$-mKate2 as described above to produce 3610 *ΔytvA ΔrsbRA ΔrsbRC ΔrsbRD amyE*::$P_{hyperspank}$-mKate2.

CSS411

MTC1693 was transduced with lysate from PY79 containing constitutive *amyE*::$P_{hyperspank}$-mKate2 as described above to produce 3610 *ΔytvA ΔrsbRA ΔrsbRB ΔrsbRD amyE*::$P_{hyperspank}$-mKate2.

CSS412

MTC1691 was transduced with lysate from PY79 containing constitutive *amyE*::$P_{hyperspank}$-mKate2 as described above to produce 3610 *ΔytvA ΔrsbRA ΔrsbRB ΔrsbRC amyE*::$P_{hyperspank}$-mKate2.

CSS414

MTC1672 was transduced with lysate from PY79 containing constitutive *amyE*::$P_{hyperspank}$-GFP as described above to produce 3610 *ΔytvA amyE*::$P_{hyperspank}$-GFP.

CSS415

MTC1697 was transduced with lysate from PY79 containing constitutive *amyE*::$P_{hyperspank}$-GFP as described above to produce 3610 *ΔytvA ΔrsbRB ΔrsbRC ΔrsbRD amyE*::$P_{hyperspank}$-GFP.

CSS416

MTC1695 was transduced with lysate from PY79 containing constitutive *amyE*::$P_{hyperspank}$-GFP as described above to produce 3610 *ΔytvA ΔrsbRA ΔrsbRC ΔrsbRD amyE*::$P_{hyperspank}$-GFP.

CSS417

MTC1693 was transduced with lysate from PY79 containing constitutive *amyE*::$P_{hyperspank}$-GFP as described above to produce 3610 *ΔytvA ΔrsbRA ΔrsbRB ΔrsbRD amyE*::$P_{hyperspank}$-GFP.

CSS418

MTC1691 was transduced with lysate from PY79 containing constitutive *amyE*::$P_{hyperspank}$-GFP as described above to produce 3610 *ΔytvA ΔrsbRA ΔrsbRB ΔrsbRC amyE*::$P_{hyperspank}$-GFP.

CSS1258

MTC1697 was transduced with a lysate from a phage from PY79 *pminiMAD-rsbRD/A* as described above to produce 3610 *ΔytvA ΔrsbRB ΔrsbRC ΔrsbRD rsbRA::RsbRD/A*. This strain was then transduced again with a constitutive *amyE*::$P_{hyperspank}$-mKate2 reporter as described above.

CSS1261

MTC1695 was transduced with a lysate from a phage from PY79 *pminiMAD-rsbRA/B* as described above to produce 3610 *ΔytvA ΔrsbRA ΔrsbRC ΔrsbRD rsbRB::RsbRA/B*. This strain was then transduced again with a constitutive *amyE*::$P_{hyperspank}$-mKate2 reporter as described above.

CSS1264

MTC1693 was transduced with a lysate from a phage from PY79 *pminiMAD-rsbRB/C* as described above to produce 3610 *ΔytvA ΔrsbRA ΔrsbRB ΔrsbRD rsbRC::RsbRB/C* This strain was then transduced again with a constitutive *amyE*::$P_{hyperspank}$-mKate2 reporter as described above.

CSS1267

MTC1695 was transduced with a lysate from a phage from PY79 *pminiMAD-rsbRC/B* as described above to produce 3610 *ΔytvA ΔrsbRA ΔrsbRC ΔrsbRD rsbRA::RsbRC/B*. This strain was then transduced again with a constitutive *amyE*::$P_{hyperspank}$-mKate2 reporter as described above.

CSS2066

MTC1697 was transduced with a lysate from a phage from PY79 *pminiMAD-ΔsigB* as described above to produce 3610 Δ*sigB ΔytvA ΔrsbRB ΔrsbRC ΔrsbRD*. This strain was then transduced again with a constitutive *amyE*::$P_{hyperspank}$-mKate2 reporter as described above.

CSS2078

MTC1691 was transduced with a lysate from a phage from PY79 *pminiMAD-ΔsigB* as described above to produce 3610 Δ*sigB ΔytvA ΔrsbRA ΔrsbRB ΔrsbRC*. This strain was then transduced again with a constitutive *amyE*::$P_{hyperspank}$-GFP reporter as described above.

MTC1683

Transformed a 3610 Δ*ytvA* recipient (MTC1672) with pminiMAD-Δ*rsbRA* (from an intermediate strain in PY79) by phage transduction, then deleted *rsbRA* via the procedure described above.

MTC1973

Strain 3610 *hag*$_{A233V}$ Δ*ytvA* Δ*rsbRA* *ywrK*::*DG1730*-P$_{rsbV}$-*mNeonGreen* (unarchived strain) was transduced with *ywrK*::P$_{hyperspank}$-mKate2 phage from JRR227 and selected on chlor.

References

1. Hamm CW, Butler DR, Cabeen MT. 2022. Bacillus subtilis Stressosome Sensor Protein Sequences Govern the Ability To Distinguish among Environmental Stressors and Elicit Different σB Response Profiles. mBio 13:e0200122.
